# Validation Strategy for Pulmonary Vein Isolation in Patients With Paroxysmal Atrial Fibrillation in Long-Term Maintaining Sinus Rhythm: A Randomized Controlled Study

**DOI:** 10.1155/2024/3672210

**Published:** 2024-10-15

**Authors:** Xinyu Li, Houdeng Yu, Shihuang Lai, Yaqi Liao, Yihong Yang, Kejun Tian, Yiming Zhong, Xinguang Chen

**Affiliations:** Division of Cardiology, The First Affiliated Hospital of Gannan Medical University, Ganzhou, China

**Keywords:** loose validation, paroxysmal atrial fibrillation, pulmonary vein isolation, rigorous validation, validation strategy

## Abstract

**Background:** Data comparing the outcomes of loose versus rigorous validation strategies for pulmonary vein isolation (PVI) in patients with paroxysmal atrial fibrillation (PAF) are limited. We aimed to prospectively assess the effectiveness of loose versus rigorous validation for PVI in patients with PAF with a maintained sinus rhythm.

**Methods:** Patients (*n* = 117) with PAF were randomized to receive either loose validation (*n* = 59) or rigorous validation (*n* = 58) after PVI. The presence of dormant conduction in loose validation was assessed only by adenosine administration followed by isoproterenol infusion. The complete absence of pulmonary vein (PV) potentials in rigorous validation was confirmed by the combination of the Lasso catheter with isoproterenol plus adenosine. Dormant conduction, revealed by validation after PVI, was ablated until all reconnections were eliminated.

**Results:** The procedure time in the rigorous validation group was greater than that in the loose validation group (161.3 ± 52.7 min vs. 142.5 ± 37.6 min, *p*=0.03, respectively). After successful PVI, the detection of dormant PV reconnections in the rigorous validation group was significantly greater than that in the loose validation group (69.0% vs. 37.3%, *p*=0.001). However, after reisolation of the sites of dormant PV conduction, the postablation recurrence rates in 1.3 years were similar between the groups (79.2% vs. 83.6%, *p*=0.67).

**Conclusion:** Rigorous validation can reveal dormant conduction in more than two-thirds of patients with PAF undergoing PVI. However, rigorous validation and additional ablation of the resulting connections do not improve long-term outcomes when a protocol that includes electrophysiological confirmation and pharmacological validation is used.

## 1. Introduction

Atrial fibrillation (AF) is the most common cardiac rhythm disturbance, and catheter ablation has become a curative therapy for maintaining sinus rhythm in patients with AF [1–3]. Anatomical and electrophysiological ablative strategies for pulmonary vein isolation (PVI) are effective for the treatment of paroxysmal AF (PAF) with catheter ablation procedures [4–6]. However, approximately 10%–30% of patients with PAF who undergo single–catheter ablation experience AF recurrence, and the percentage of patients with persistent AF is even greater [7–10]. Increasing evidence suggests that pulmonary vein (PV) reconnection or reconduction is the most common reason for recurrent atrial tachyarrhythmia after complete circular PVI [11–13]. Additional ablation procedures targeting recovered PV conduction have shown to potentially increase single ablation success rates and improve long-term outcomes [14]. Therefore, achieving a durable, reliable, and complete PVI is one of the ultimate goals pursued by physicians. Two validation strategies have been described for the detection of PV reconnection over previously isolated PVs. One strategy is based on pharmacology to reveal dormant PV conduction. Adenosine alone, isoproterenol administration, or adenosine injection during isoproterenol infusion has been used to reveal latent PV reconnection after PVI [15–20]. The other strategy involves applying electrophysiological methods to confirm the absence of dormant conduction. The verification of PVI bidirectional conduction blocks (entrance and exit blocks) via a PV mapping catheter is the most common practice. The pace-and-ablate technique, which is based on simultaneous pacing and ablation through the tip of a single mapping/ablation catheter, is applicable for electrically proven PVI bidirectional conduction blocks [21, 22]. Alternatively, complete loss of pace capture along the circumferential ablation line surrounding the PVs can also be used to identify conduction gaps after ablation of the initial lesions [23]. There is variability in PVI validation with the use of different strategies. The data suggest that pharmacological validation via loose PVI validation strategies, such as adenosine tests after PVI, to confirm the absence of dormant conduction and triggers initiating AF is beneficial for improving outcomes after catheter ablation of PAF [17]. A previous study demonstrated that rigorously confirming complete PV electrical isolation was superior to purely anatomical techniques of PVI [24]. However, data comparing the outcomes of loose versus rigorous validation strategies for PVI are limited. The most rigorous electrophysiological validation strategy for PVI is the demonstration of bidirectional conduction blocks. We developed a rigorous PVI validation technique in which electrophysiological confirmation of PV disconnection is combined with pharmacological validation of PVI. The aim of the present study was to prospectively assess the effectiveness of loose versus rigorous validation strategies for PVI in patients with PAF in long-term maintaining sinus rhythm.

## 2. Methods

### 2.1. Study Design

**Figure 1:**
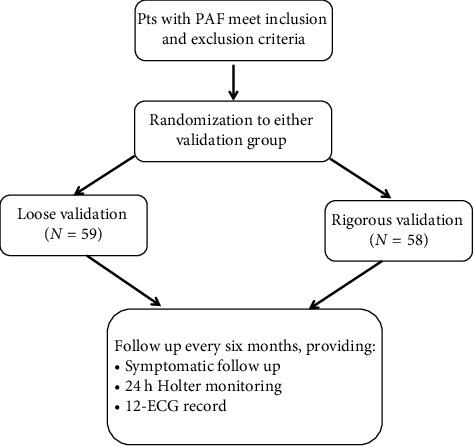
Study flowchart. PAF: paroxysmal AF.

This study was a prospective, single-center, randomized, 2-arm trial that was performed in patients with PAF who underwent initial catheter ablation. This study was approved by the ethics committee. Patients were recruited at our institution from March 2018 to May 2021 and randomized in a 1:1 fashion. Randomization to either study arm was conducted before the procedure on the basis of a previously generated computer algorithm. The patients were blinded to the ablation strategy but the operators were not. The study was conducted according to the guidelines of the Declaration of Helsinki and approved by the Institutional Review Board of the First Affiliated Hospital of Gannan Medical University (LLSL-2018235). Written informed consent was obtained from each patient prior to the PV ablation procedure (Figure 1).

### 2.2. Study Population

Patients were eligible for the study if they (1) had a history of symptomatic PAF (defined as self-terminating PAF within 7 days of onset), (2) were aged 18 to 75 years, and (3) were able and willing to provide written informed consent. The detailed exclusion criteria were as follows: (1) intracardiac thrombus, (2) left atrial (LA) size > 50 mm, (3) previous ablation for AF, (4) cerebral infarction acute phase, (5) hematological system disease, (6) severe liver and kidney dysfunction, (7) pregnancy or lactation, (8) life expectancy < 12 months, (9) psychopathy, and (10) severe structural cardiac disease (severe mitral regurgitation, dilated cardiomyopathy, hypertrophic cardiomyopathy, and other severe valvular heart diseases).

### 2.3. Electrophysiological Study

All antiarrhythmic drugs (AADs) except amiodarone were discontinued for more than five half-lives prior to the ablation procedure. The entire procedure was performed while the patient was in a conscious or deep sedated state. All patients received effective anticoagulation for at least 1 month. Before the procedure, transesophageal echocardiography or contrasted computed tomography was performed to exclude intracardiac thrombus. A 6F decapolar catheter (St. Jude Medical, Inc., St. Paul, Minnesota, United States of America) was positioned in the coronary sinus via the left femoral vein to perform electrogram recordings and obtain a system positional reference. A 6F quadripolar catheter (St. Jude Medical, Inc.) was advanced into the right ventricle via the femoral vein. Two 8F long sheaths (SL1, St. Jude Medical, Inc.) were advanced to the LA through standard transseptal puncture. A deflectable decapolar circular catheter (Lasso catheter, Biosense Webster) was advanced through the sheath for PV mapping, and a deflectable quadripolar open irrigated catheter (IBI, St. Jude Medical, Inc.) was inserted into the LA for mapping and ablation. After transseptal puncture, intravenous heparin was administered to maintain an activated clotting time of 250–300 s. The activated clotting time was monitored every 30 min, and the heparin dose was adjusted accordingly. Intracardial electrograms were recorded via a digital electrophysiological recording system (Prucka CardioLab, General Electric Health Care System Inc., Milwaukee, Wisconsin, United States of America) and were filtered from 30 to 300 Hz.

### 2.4. Mapping and Catheter Ablation

After catheter placement, the electroanatomical geometries of the LA and PVs were constructed via a circular mapping and/or an ablation catheter with an EnSite-NavX (St. Jude Medical, Inc.) or CARTO (Biosense Webster Inc.) mapping system. The PV ostia were mapped and tagged onto the electroanatomical map guided by pulmonary venography, 3D mode, and local potentials. The techniques used for PVI have been previously described [25]. In brief, prior to ablation, the circular mapping catheter was placed sequentially within each PV antra to record PV potentials. Circumferential–antral ablation was performed approximately 1 cm outside of the ostium of both the left and right PVs to encircle and electrically isolate each PV antrum. As each antrum was encircled, a circular mapping catheter was used to confirm electrical isolation. During the ablation procedure, the circular mapping catheter was positioned in the ipsilateral upper PV to record the electrical activity of the PV. After the initial ablation procedure with documented electrical isolation of the upper PV, the circular mapping catheter was then positioned at the lower PV to confirm the presence of PV isolation. Once all PV potentials recorded by the circular mapping catheter within each antrum were abolished, isolation of the PV antrum was considered complete. The power was set at 30 to 35 W with irrigation rates of 5 to 20 mL/min to achieve the desired power delivery during ablation. When ablations were performed on the posterior wall of the LA, the power was reduced to 30 W. RF energy was delivered for 15 to 20 s at each point. The target ablation index was 500 for the anterior walls, whereas that for the posterior walls was 350–400. The temperature was limited to 45°C.

### 2.5. Loose Validation for PVI

Patients randomized to this arm only received pharmacological validation after completion of the circumferential–antral ablation lesion set. After PVI was complete, dormant conduction was sequentially assessed for each PV in response to intravenous injection of adenosine followed by isoproterenol infusion. Isoproterenol infusion was initiated approximately two minutes after PVI was completed, with a target heart rate > 100 bpm or a maximum dose of 20 *μ*g/min. Once the target heart rate was achieved during isoproterenol infusion, we performed provocative testing with at least 10 mg bolus of endovenous adenosine separately and sequentially for each PV utilizing the circular mapping catheter. The adenosine dose was titrated to achieve at least one blocked P wave or a sinus pause for 3 s or more. Dormant conduction was defined as the reappearance of PV conduction recorded on the circular mapping catheter for one beat or more. Additional RF applications were performed to eliminate dormant conduction in each affected PV until all PV reconnections were confirmed to be absent.

### 2.6. Rigorous Validation for PVI

Patients in this group underwent electrophysiological confirmation in combination with pharmacological validation. Patients randomized to this arm first underwent electrophysiological examination to verify whether complete entrance and exit blocks were achieved. After anatomical isolation of all the PVs was achieved, the complete bidirectional block across the ablation lines was confirmed via the Lasso catheter. The entrance block was demonstrated by the loss of PV potentials on all poles of the circumferential catheter positioned at the entrance of the PV, and the exit block was demonstrated by failure to capture the LA by pacing (at 10 mA and 2 ms pulse width) each of the 10 bipolar pairs of electrodes of the circumferential catheter positioned at the entrance of the PV. If the LA was still captured by pacing, additional ablation lesions were created until atrial capture was no longer possible at that location. Once complete bidirectional block confirmation was achieved, isoproterenol plus adenosine was used to provoke dormant PV conduction as described in the loose validation group with the same protocol. We also performed additional ablation in cases of dormant conduction or trigger sites of pharmaceutical-induced AF.

## 3. Outcomes

The primary endpoint was the time to first recurrence of symptomatic electrocardiographically documented atrial tachyarrhythmia (AF, atrial flutter, or atrial tachycardia) during follow-up after a single-ablation procedure. A repeat ablation procedure performed at any time for any atrial tachyarrhythmia, which was also considered as a primary endpoint event. An atrial tachyarrhythmia qualified as a recurrence if it lasted 30 s or longer and was documented by a 12-lead electrocardiogram, electrocardiographic rhythm strips, or transtelephonic monitor recordings.

### 3.1. Follow-Up

For the purpose of the study, we chose a follow-up time of 24 months or as close to 24 months as possible for the data collection. The patients resumed their AADs after the procedure but then stopped after a 3-month postablation blanking period. Recurrences during the 3-month blanking period were treated with AADs and/or cardioversion if needed. Other prescribed drugs, including antihypertensives and statins, were continued during the follow-up period. All patients underwent a routine follow-up examination at our outpatient clinic 2 weeks after ablation and then at 1 month and every 1–3 months thereafter. Twenty-four-hour Holter recordings were scheduled at 3, 6, 12, 18, and 24 months after ablation. Successful ablation was defined as nonrecurrence of AF lasting more than 30 s on a standard ECG or 24-h Holter recording during the follow-up period after the 3-month postablation blanking period.

### 3.2. Statistical Analysis

We present continuous variables as the means and SDs or medians and IQRs according to whether the distribution was normal or non-normal. Differences in continuous variables between the two groups were analyzed by an unpaired *t* test or the Mann‒Whitney *U* test. We present categorical variables as percentages and compared them via *χ*^2^ tests or Fisher's exact tests. We checked the statistical assumptions before the analysis. All the statistical tests and confidence intervals were two-sided, with a significance level of 0.05.

## 4. Results

### 4.1. Baseline Characteristics

The baseline characteristics of the 117 patients included in the study are presented in Table 1. Fifty-nine patients (50.4%) were randomly assigned to the loose validation group, and 58 patients (49.6%) were assigned to the rigorous validation group. All the PVs were completely isolated in all the patients. There were no significant differences in the clinical characteristics or in the LAD or LVEF between the groups.

### 4.2. Procedural Findings

Table 2 shows that there was no significant difference in X-ray time, X-ray dose, RPV RF time, or LPV RF time between the two groups. However, the procedure time in the rigorous validation group was greater than that in the loose validation group (161.3 ± 52.7 min vs. 142.5 ± 37.6 min, *p*=0.03, respectively). After successful PVI, the percentage of patients with dormant PV reconnections in the rigorous validation group was significantly greater than that in the loose validation group (40 (69.0%) of 58 patients vs. 22 (37.3%) of 59 patients, *p*=0.001). In the loose validation group, the number of PV reconnection sites was 1 PV site in 7 (11.9%) patients, 2 PV sites in 11 (18.6%), and 3 PV sites in 4 (6.8%). In the rigorous validation group, the number of PV reconnection sites was 1 PV site in 17 (11.9%) patients, 2 PV sites in 16 (27.6%), and 3 PV sites in 7 (12.1%) patients (*p*=0.006). Overall, the median number of dormant PV reconnection sites was significantly greater in the rigorous validation group than in the loose validation group (Table 3). The distribution of dormant PV conduction sites per group is shown in Table 3. In the loose validation group, dormant PV conduction occurred at 14 (34.1%) sites in the RSPV, 12 (29.3%) sites in the RIPV, 8 (19.5%) in the LSPV, and 7 (17.1%) sites in the LIPV. In the rigorous validation group, dormant PV conduction occurred at 21 (30%) sites in the RSPV, 24 (34.3%) sites in the RIPV, 20 (28.6%) in the LSPV, and 5 (7.1%) sites in the LIPV. However, there was no significant difference in the distribution of dormant PV sites between the two groups (*p*=0.33).

### 4.3. Follow-Up

During a mean follow-up period of 16.5 ± 6.0 months, eleven (9.4%) patients were lost, of whom, 6 (10.2%) were in the loose validation group and 5 (8.6%) were in the rigorous validation group. After the first 3-month blanking period, there was no difference in the rate of AF recurrence in patients who received rigorous validation (8/53, 15.1%) compared with those who received loose validation (10/53; 18.9%; *p*=0.61).

### 4.4. Complications

During the procedure, 6 patients in the loose validation group and 9 patients in the rigorous validation group experienced vagal responses. Vagal responses were frequently observed during ablation in the LSPV (100%), and all patients experienced complete resumption via continuous temporary pacing and/or the use of vasopressor agents until the end of the procedure. Cardiac perforation occurred in only one patient in the rigorous validation group. After pericardial puncture, the patient was no longer in danger and was discharged alive. One patient in the rigorous validation group experienced hypothyroidism due to amiodarone use during the follow-up period.

## 5. Discussion

### 5.1. Major Findings

In the present study, we assessed the differential efficacy of rigorous validation and loose validation to reveal dormant PV conduction in patients with PAF who were undergoing PVI. We found that rigorous validation was superior to loose validation in revealing dormant conduction, but the procedure time of rigorous validation was longer than that of loose validation. However, there was no difference in arrhythmia recurrence between patients who underwent additional RF to eliminate PV reconnections guided by a rigorous validation strategy and those who underwent additional RF guided by a loose validation strategy.

Owing to recurrent AF associated with PV reconnection, the success rate of a single RF procedure for PAF is limited, and targeted ablation at reconnected PV sites may reduce the risk of subsequent AF recurrence [26]. Theoretically, the identification of concealed PV reconnection with different strategies at the time of the initial intervention, followed by the application of ablation for additional RF lesions to eliminate reconnection, may achieve durable PVI and prevent AF recurrence. Two strategies for the early detection of PV reconnection after PVI were adopted in the present study. One strategy consisted of pharmacological approaches, including adenosine and isoproterenol. Adenosine or isoproterenol is used clinically to reveal dormant PV conduction and guide ablation of additional lesions [15, 27]. In our loose validation group, the combination of adenosine and isoproterenol resulted in dormant conduction in 37.3% of the patients with PAF who underwent PVI, which was analogous to the findings of a previously reported study [28]. The other strategy is based on LA capture to reveal the likelihood of reconnection. The methodology involves achieving an unexcitable ablation line by pacing along the ablation line, and further performing ablation until a loss of LA capture is achieved. Previous studies have demonstrated that pace capture along the ablation line can be used to identify conduction gaps, even gaps that would have not been detected by testing for dormant conduction [21, 29]. In our study, rigorous validation could detect more dormant conduction, so more RF ablation energy was required to achieve loss of pace capture along the ablation line. However, the procedure duration was significantly longer in the rigorous validation group, which was consistent with the findings of a previous study [30].

The success rate 1.3 years after AF ablation was 84.9% in our rigorous validation group, which was similar to that reported in a randomized study conducted by Steven et al., in which the success rate at 1 year was 82.7% in their pace-and-ablate group [30]. Moreover, after a mean follow-up of 1.3 years, 81.1% of the patients in our control group were free from any AF/AT after a single procedure, and the results were analogous to those of a previous study [31]. Although Steven et al. reported the use of pacing to ensure an unexcitable gap along the PVI line, in comparison with the conventional ablation group, the short-term single ablation success rate has markedly increased [30]. Compared with the loose validation method, the creation of durable lesions guided by the rigorous validation method may have a beneficial effect on the outcome after AF ablation, but our study failed to show a significant improvement in the outcome after ablation via this method. There are several possible reasons for this result. First, the success rate in our control group was 81.1%, which was higher than that in the corresponding conventional ablation group in Steven's study, with only 52% of the patients being free from AF recurrence. Therefore, the beneficial effect of the rigorous validation method on post-PVI outcomes may be small compared with that of the control method, which has a high success rate. In addition, the relatively small study group may have influenced the results of our statistical analyses. Further prospective, large, randomized studies are needed to clarify whether the loose validation method and rigorous validation method have true beneficial effects on the outcome of PAF ablation.

Sites of reconnection were observed in 69.0% of the patients in the rigorous validation group. Pacing at the ablation line enables the detection of potential recovery sites independent of actual reconnection. Therefore, pacing seems to identify possible recovery sites at the ablation line more precisely. Nonetheless, the overall distribution of sites of PV reconnection in the rigorous validation group was similar to that in the loose validation group, suggesting that the PV reconnections may be due mainly to inadequate tissue surface–catheter tip contact or difficulties in catheter manipulation due to the patient's anatomy. Interestingly, the prevalence of dormant conduction was greater for right-sided PVs than for left-sided PVs in our study. This complex phenomenon can be attributed to anatomical and physiological factors. First, the right PVs tend to have a more direct and extensive connection with the atrium, especially the superior vena cava (SVC), making it easier for electrical signals to propagate between these structures. The SVC passes immediately adjacent to the RSPV, and anatomical and electrophysiological relationships could be present between the two structures [32, 33]. Second, we cannot completely exclude that far-field electrograms recorded at the SVC or RSPV could result in a misdiagnosis of the origin of arrhythmic activities.

### 5.2. Study Limitations

Our study had several limitations. First, our methods used for postablation follow-up monitoring may have underestimated the incidence of AF recurrence after ablation. However, AF recurrence in both groups was determined by the same follow-up methods, which may have minimized the effect when comparing the outcomes. Second, because pacing at a site may capture a wider area than expected, pacing along the ablation lines may capture sites beyond the ablation lines or other unintended sites. To prevent this, however, the Lasso catheter was carefully placed along the stable ablation line, and unipolar pacing was used to capture the local site. Third, the use of a single Lasso catheter requires subsequent PV testing for dormant connections. The use of 2 Lasso catheters could deliver simultaneous information in ipsilateral PVs and more precise information regarding the exact dormant connection site. Furthermore, only one pacing current output was used. Increasing the pacing output may reveal the occurrence of dormant PV conduction.

## 6. Conclusion

Rigorous validation can reveal dormant conduction in more than two-thirds of patients with PAF who undergo PVI. However, rigorous validation and additional ablation of the resulting connections do not improve long-term outcomes with the use of a protocol that includes electrophysiological confirmation and pharmacological validation.

## Figures and Tables

**Table 1:** Baseline characteristics.

Variable	Loose group (*N* = 59)	Rigorous group (*N* = 58)	*p* value
Age (yrs)	60.2 ± 9.7	61.0 ± 10.2	0.67
Females (%)	22 (37.3%)	21 (36.2%)	0.90
Height (cm)	161.8 ± 23.3	164.1 ± 23.6	0.59
Weight (kg)	67.3 ± 10.9	69.9 ± 10.2	0.19
Smoke (%)	25 (42.4%)	27 (47.4%)	0.59
Drink (%)	28 (47.5%)	24 (42.1%)	0.56
Hypertension (%)	23 (39.7%)	32 (56.1%)	0.08
Diabetes (%)	0 (0%)	3 (5.3%)	0.12
CAD (%)	6 (10.2%)	4 (7.1%)	0.81
Stroke history (%)	2 (3.4%)	8 (14.0%)	0.09
Thyroid disease (%)	3 (5.2%)	1 (1.8%)	0.62
AADs use	51 (86.4%)	52 (91.2%)	0.41
LAD (cm)	35.9 ± 4.0	36.8 ± 4.8	0.32
LVDD (cm)	47.2 ± 5.2	47.5 ± 3.8	0.73
LVDs (cm)	32.8 ± 7.7	30.9 ± 2.2	0.15
LVEF (%)	61.9 ± 7.1	64.0 ± 3.6	0.06
LAV(mm^3^)	94.2 ± 31.1	99.3 ± 24.2	0.32
AI tool (%)	30 (50.8%)	32 (55.2%)	0.64
CHADS_2_VASc	1.9 ± 0.7	2.1 ± 0.8	0.09

*Note:* The values are the mean ± SD, median (interquartile range), or *n* (percent).

Abbreviations: AADs, antiarrhythmic drugs; AI, ablation index; CAD, coronary atherosclerotic disease; LAD, left atrial diameter; LAV, left atrium volume; LVDD, left ventricular end-diastolic diameter; LVDs, left ventricular end systolic diameter; LVEF, left ventricular ejection fraction.

**Table 2:** Procedure parameters.

	Loose group (*N* = 59)	Rigorous group (*N* = 58)	*p* value
Procedure time (min)	142.5 ± 37.6	161.3 ± 52.7	0.03
X-ray time (min)	17.9 ± 6.4	18.3 ± 7.7	0.77
X-ray dose (mGy)	138.7 ± 95.0	140.9 ± 88.6	0.90
RPV RF time (min)	18.5 ± 6.6	20.3 ± 8.7	0.22
LPV RF time (min)	20.4 ± 11.4	21.2 ± 8.2	0.67
Recurrence (> 3 month) (%)	10 (18.9%)	8 (15.1%)	0.61
Follow-up time (month)	17 ± 6.1	15.9 ± 5.8	0.38
Complication (%)	9 (15.3%)	9 (15.5%)	0.97

*Note:* The values are the mean ± SD, or *n* (percent).

Abbreviations: LPV, left PV; RF, radiofrequency; RPV, right PV.

**Table 3:** Numbers of patients with dormant PV reconnections and the distribution of the PV reconduction sites.

	Loose group (*N* = 59)	Rigorous group (*N* = 58)	*p* value
No. of patients with PV reconnection	22 (37.3%)	40 (69.0%)	0.001
PV reconnection no.			0.006
0	37 (62.7%)	18 (31.0%)	
1	7 (11.9%)	17 (11.9%)	
2	11 (18.6%)	16 (27.6%)	
3	4 (6.8%)	7 (12.1%)	
Median no. of PV reconnections	0 (0, 2)	1 (0, 2)	0.003
Distribution of the PV reconduction sites	41	70	0.33
RSPV	14 (34.1%)	21 (30.0%)	
RIPV	12 (29.3%)	24 (34.3%)	
LSPV	8 (19.5%)	20 (28.6%)	
LIPV	7 (17.1%)	5 (7.1%)	
RPV	26 (63.4%)	45 (64.3%)	0.93
LPV	15 (36.6%)	25 (35.7%)	

*Note:* The values are the *n* (percent) or median (interquartile range).

Abbreviations: LIPV, left inferior PV; LPV, left PV; LSPV, left superior PV; PV, pulmonary vein; RIPV, right inferior PV; RPV, right PV; RSPV, right superior PV.

## Data Availability

The data used to support the findings of this study are available from the corresponding author upon reasonable request.

## References

[1] January C. T., Wann L. S., Calkins H. (2019). 2019 AHA/ACC/HRS Focused Update of the 2014 AHA/ACC/HRS Guideline for the Management of Patients With Atrial Fibrillation: A Report of the American College of Cardiology/American Heart Association Task Force on Clinical Practice Guidelines and the Heart Rhythm Society in Collaboration With the Society of Thoracic Surgeons. *Circulation*.

[2] Mariani M. V., Pierucci N., Piro A. (2022). Incidence and Determinants of Spontaneous Cardioversion of Early Onset Symptomatic Atrial Fibrillation. *Medicina*.

[3] La Fazia V. M., Pierucci N., Mohanty S. (2023). Catheter Ablation Approach and Outcome in HIV+ Patients With Recurrent Atrial Fibrillation. *Journal of Cardiovascular Electrophysiology*.

[4] Kuck K. H., Hoffmann B. A., Ernst S. (2016). Impact of Complete Versus Incomplete Circumferential Lines Around the Pulmonary Veins During Catheter Ablation of Paroxysmal Atrial Fibrillation: Results From the Gap-Atrial Fibrillation-German Atrial Fibrillation Competence Network 1 Trial. *Circulation: Arrhythmia and Electrophysiology*.

[5] Müller J., Nentwich K., Berkovitz A. (2023). Recurrent Atrial Fibrillation Ablation After Initial Successful Pulmonary Vein Isolation. *Journal of Clinical Medicine*.

[6] Serban T., Mannhart D., Abid Q. U. (2023). Durability of Pulmonary Vein Isolation for Atrial Fibrillation: A Meta-Analysis and Systematic Review. *Europace*.

[7] Wilber D. J., Pappone C., Neuzil P. (2010). Comparison of Antiarrhythmic Drug Therapy and Radiofrequency Catheter Ablation in Patients With Paroxysmal Atrial Fibrillation: A Randomized Controlled Trial. *JAMA*.

[8] Cosedis Nielsen J., Johannessen A., Raatikainen P. (2012). Radiofrequency Ablation as Initial Therapy in Paroxysmal Atrial Fibrillation. *New England Journal of Medicine*.

[9] Şaylık F., Çınar T., Akbulut T., Hayıroğlu M. İ. (2023). Comparison of Catheter Ablation and Medical Therapy for Atrial Fibrillation in Heart Failure Patients: A Meta-Analysis of Randomized Controlled Trials. *Heart & Lung*.

[10] Poole J. E., Bahnson T. D., Monahan K. H. (2020). Recurrence of Atrial Fibrillation After Catheter Ablation or Antiarrhythmic Drug Therapy in the CABANA Trial. *Journal of the American College of Cardiology*.

[11] Ouyang F., Antz M., Ernst S. (2005). Recovered Pulmonary Vein Conduction as a Dominant Factor for Recurrent Atrial Tachyarrhythmias after Complete Circular Isolation of the Pulmonary Veins: Lessons from Double Lasso Technique. *Circulation*.

[12] Gerstenfeld E. P., Callans D. J., Dixit S., Zado E., Marchlinski F. E. (2003). Incidence and Location of Focal Atrial Fibrillation Triggers in Patients Undergoing Repeat Pulmonary Vein Isolation: Implications for Ablation Strategies. *Journal of Cardiovascular Electrophysiology*.

[13] Nanthakumar K., Plumb V. J., Epstein A. E., Veenhuyzen G. D., Link D., Kay G. N. (2004). Resumption of Electrical Conduction in Previously Isolated Pulmonary Veins: Rationale for a Different Strategy?. *Circulation*.

[14] Wang X. H., Liu X., Sun Y. M. (2007). Early Identification and Treatment of PV Re-Connections: Role of Observation Time and Impact on Clinical Results of Atrial Fibrillation Ablation. *Europace*.

[15] Hachiya H., Hirao K., Takahashi A. (2007). Clinical Implications of Reconnection Between the Left Atrium and Isolated Pulmonary Veins Provoked by Adenosine Triphosphate After Extensive Encircling Pulmonary Vein Isolation. *Journal of Cardiovascular Electrophysiology*.

[16] Tokuda M., Matsuo S., Isogai R. (2016). Adenosine Testing During Cryoballoon Ablation and Radiofrequency Ablation of Atrial Fibrillation: A Propensity Score-Matched Analysis. *Heart Rhythm*.

[17] Iqbal M., Jena A., Park H. S. (2017). Value of Adenosine Test to Reveal Dormant Conduction or Adenosine-Induced Atrial Fibrillation after Pulmonary Vein Isolation. *J Arrhythm*.

[18] Kobori A., Shizuta S., Inoue K. (2015). Adenosine Triphosphate-Guided Pulmonary Vein Isolation for Atrial Fibrillation: the UNmasking Dormant Electrical Reconduction by Adenosine TriPhosphate (UNDER-ATP) Trial. *European Heart Journal*.

[19] Blandino A., Biondi-Zoccai G., Battaglia A. (2017). Impact of Targeting Adenosine-Induced Transient Venous Reconnection in Patients Undergoing Pulmonary Vein Isolation for Atrial Fibrillation: A Meta-Analysis of 3524 Patients. *Journal of Cardiovascular Medicine*.

[20] Tutuianu C., Pap R., Riesz T., Bencsik G., Makai A., Saghy L. (2019). Is Adenosine Useful for the Identification of Atrial Fibrillation Triggers?. *Journal of Cardiovascular Electrophysiology*.

[21] Steven D., Reddy V. Y., Inada K. (2010). Loss of Pace Capture on the Ablation Line: A New Marker for Complete Radiofrequency Lesions to Achieve Pulmonary Vein Isolation. *Heart Rhythm*.

[22] Kato K., Hasegawa S., Kikuchi S. (2022). Impact of High Frequency Stimulation to Confirm a Complete Box Isolation in Catheter Ablation of Non-Paroxysmal Atrial Fibrillation. *Pacing and Clinical Electrophysiology*.

[23] Eitel C., Hindricks G., Sommer P. (2010). Circumferential Pulmonary Vein Isolation and Linear Left Atrial Ablation as a Single-Catheter Technique to Achieve Bidirectional Conduction Block: The Pace-And-Ablate Approach. *Heart Rhythm*.

[24] Mantovan R., Verlato R., Calzolari V. (2005). Comparison Between Anatomical and Integrated Approaches to Atrial Fibrillation Ablation: Adjunctive Role of Electrical Pulmonary Vein Disconnection. *Journal of Cardiovascular Electrophysiology*.

[25] Hocini M., Jaïs P., Sanders P. (2005). Techniques, Evaluation, and Consequences of Linear Block at the Left Atrial Roof in Paroxysmal Atrial Fibrillation: A Prospective Randomized Study. *Circulation*.

[26] Cappato R., Negroni S., Pecora D. (2003). Prospective Assessment of Late Conduction Recurrence across Radiofrequency Lesions Producing Electrical Disconnection at the Pulmonary Vein Ostium in Patients with Atrial Fibrillation. *Circulation*.

[27] Matsuo S., Yamane T., Date T. (2010). Comparison of the Clinical Outcome after Pulmonary Vein Isolation Based on the Appearance of Adenosine-Induced Dormant Pulmonary Vein Conduction. *American Heart Journal*.

[28] Ghanbari H., Jani R., Hussain-Amin A. (2016). Role of Adenosine After Antral Pulmonary Vein Isolation of Paroxysmal Atrial Fibrillation: A Randomized Controlled Trial. *Heart Rhythm*.

[29] Schaeffer B., Willems S., Sultan A. (2015). Loss of Pace Capture on the Ablation Line During Pulmonary Vein Isolation Versus “Dormant Conduction”: Is Adenosine Expendable?. *Journal of Cardiovascular Electrophysiology*.

[30] Steven D., Sultan A., Reddy V. (2013). Benefit of Pulmonary Vein Isolation Guided by Loss of Pace Capture on the Ablation Line: Results from a Prospective 2-center Randomized Trial. *Journal of the American College of Cardiology*.

[31] Okumura Y., Watanabe I., Nagashima K. (2014). The Effects of Standard Electrical PV Isolation vs. “Pace and Ablate” on ATP-Provoked PV Reconnections. *Journal of Interventional Cardiac Electrophysiology*.

[32] oshida K., Hattori A., Tsuneoka H. (2017). Electrophysiological Relation Between the Superior Vena Cava and Right Superior Pulmonary Vein in Patients With Paroxysmal Atrial Fibrillation. *Journal of Cardiovascular Electrophysiology*.

[33] Yoshida K., Baba M., Hasebe H. (2019). Structural Relation Between the Superior Vena Cava and Pulmonary Veins in Patients with Atrial Fibrillation. *Heart and Vessels*.

